# Communicating antimicrobial resistance: the need to go beyond human health

**DOI:** 10.1093/jacamr/dlab096

**Published:** 2021-07-27

**Authors:** Kelly Thornber, Emma Pitchforth

**Affiliations:** 1 Department of Biosciences, University of Exeter, Stocker Road, Exeter, EX4 4QD, UK; 2 College of Medicine and Health, University of Exeter, St Luke's Campus, Heavitree Road, Exeter, EX1 2LU, UK

Despite significant resource mobilization to address antimicrobial resistance (AMR), a persistent ‘action gap’ remains between AMR governance and the realization of societal change. Across international and national governance networks, AMR is widely recognized as an issue that goes beyond human health, requiring considerable and urgent attention from all sectors of society. In contrast, AMR awareness campaigns remain predominantly focused on framing AMR as a human health issue, with messages targeting individualistic antimicrobial consumption. In this article, we make the case that AMR awareness campaigns should frame the issue as one that goes beyond human health, in order to engage a greater number and broader array of stakeholders from across society. We argue that this could support efforts to reduce the AMR action gap, by making the issue more relevant to a wider range of stakeholders, expanding the sphere of responsibility and moving the focus from individualistic to societal-wide change. 

The COVID-19 pandemic has demonstrated not only the far-reaching impact of a single untreatable infectious pathogen but also the scope for rapid, societal-wide change in response to such a threat. Antimicrobial resistance (AMR) has received considerable international attention and resources, and potentially poses a much greater long-term clinical, economic, social and environmental threat than COVID-19,^1–3^ yet societal-wide engagement and action on the issue is proving difficult to realize.^4^^,^^5^ At the heart of the response to COVID-19 has been effective communication between stakeholders from different sectors, with problems of noncompliance blamed on poor clarity and consistency of messaging, even when backed by law.^6^ The UK Government’s independent review on AMR recommended a massive global awareness campaign estimated at 40–100 million USD per year,^3^ and effective AMR communication is a cornerstone of the global AMR action plan,^7^ however studies continue to show relatively low levels of AMR awareness across the globe, with common misunderstandings and a sense among the public that AMR is an issue beyond their control.^8–10^ The Interagency Co-ordination Group (IACG) on AMR have identified the need to go beyond human health when communicating AMR, recommending it is framed in the context of achieving wider societal goals such as food security and sustainable development in order to engage publics outside the clinic and overcome the AMR public–policy ‘action gap’.^4^^,^^11^ Accordingly, the holistic One Health approach has become a dominant discourse within international AMR policy documents and national AMR action plans, despite criticism over its inherent anthropocentricity.^11–15^ In contrast, the majority of public awareness campaigns to date, and the recent guidelines developed to support these, have remained focused on framing AMR as a human health issue, with messages largely targeting individual clinical encounters and antibiotic misuse rather than the wider societal action that is required to address both antibiotic misuse and AMR transmission.^8^^,^^16^^,^^17^ For example, the UK’s ongoing ‘Keep Antibiotics Working’ campaign specifically targets individual antibiotic users, and frames AMR as a human health issue that risks ‘a more severe or longer illness’.^18^ Guidelines on the responsible use of antibiotics in UK farm animals frame AMR as an issue that could affect both human and animal health, but defer responsibility from antimicrobial use in animals, repeatedly stating that ‘The consensus amongst experts is that the main cause of antibiotic resistance in human pathogens is the overuse and/or inappropriate use of antibiotics in human medicine’.^19^ Thus, despite the acknowledged importance of inclusive framing to engage a wider audience, this is not how AMR is typically being communicated to the public. We draw upon evidence to make the case that this needs to change. 

Widespread public support has been shown to be key for both bringing policy development to the fore as well as for successful implementation,^8^ and we take the assumption that effective communication plays an important role in mobilizing the coordinated support and effort required across sectors and disciplines to achieve this.^20^^,^^21^ How a message is framed is key to clearly defining the problem, who is accountable, who will be impacted and, importantly, the solutions.^22^ Framing AMR as an issue that goes beyond human health would expand its defined impacts to incorporate the significant risks that AMR transmission and the inappropriate use of antimicrobial compounds pose to global food security, animal welfare and the natural environment. In turn, this would expand the size of the population and the breadth of stakeholders who consider the issue to have relevance to their lives, increasing the number of people and organizations who may take action to address it. More inclusive framing of AMR would also validate the use of animals and the environment to engage the public on the issue; these are two emotive subjects that could help to gain public support, and could be used to expand the impacts of visual imagery, which is particularly emotive yet traditionally challenging for AMR awareness campaigns due to the microscopic scale at which AMR operates.^8^^,^^23^ The use of antimicrobials in farm animals and the environmental impacts of pharmaceutical pollutants are current issues within the public domain and may be more immediate to some publics than resistant infections;^24^^,^^25^ incorporating these into AMR communications may therefore help to overcome our common tendencies towards temporal discounting and proximization, whereby future and more distant risks are perceived with reduced severity.^20^^,^^26^ More inclusive definitions of the causes and solutions to the issue would also widen engagement by going beyond ‘antimicrobial misuse’ and bringing greater attention to the myriad of societal-wide factors that are increasingly established as the major forces driving global AMR.^27–29^ By framing AMR as an issue that goes beyond human health, the solutions defined, and responsibility for their uptake, would reach further than the individual antimicrobial consumer, who often has limited capacity to act.^20^^,^^30^ This would extend culpability to sectors of society that may seem remote from modern medicine but nevertheless contribute towards providing an environment in which AMR can emerge and disseminate, such as the food and manufacturing industries.^31^

The international AMR community has called for more research into optimizing AMR communications for local contexts, to improve understanding of localized AMR risk in order to evaluate/prioritize interventions and tailor solutions.^4^^,^^8^^,^^10^^,^^16^^,^^32^^,^^33^ To this end, framing AMR as an issue that goes beyond human health would enable a broader range of messages to be targeted to more diverse audiences, and importantly help to engage those for whom the risk of their actions undermining modern medicine is not a priority, for example, stakeholders in food production industries in low- and middle-income countries.^30^ Future AMR awareness planning could take lessons from COVID-19 and climate change research into strategic narratives to address policy–society action gaps, which have highlighted the importance of engaging all stakeholders across society to iteratively co-create a narrative through constructive dialogue.^6^^,^^34^ As the AMR Global Leaders Group continues to provide leadership for AMR communication strategies, research into their practical implementation should engage with those for whom these strategic narratives are targeted. This needs to go beyond human health, to include the plethora of actors from across the breadth of society whose actions are responsible for the causes, solutions, and fate, of AMR.

## References

[1] Charlton E. The Looming Health Catastrophe That Could Be More Deadly than COVID-19. World Economic Forum, 2020. https://www.weforum.org/agenda/2020/11/superbugs-health-risk-antimicrobial-resistance/.

[2] The Guardian. ‘Superbugs’ a Far Greater Risk Than Covid in Pacific, Scientist Warns, 2020. https://www.theguardian.com/world/2020/sep/10/superbugs-a-far-greater-risk-than-covid-in-pacific-scientist-warns.

[3] O’Neill J. Tackling Drug-Resistant Infections Globally: Final Report and Recommendations. Review on Antimicrobial Resistance, 2016. https://wellcomecollection.org/works/thvwsuba.

[4] IACG. Meeting the Challenge of Antimicrobial Resistance: From Communication to Collective Action. 2018. https://www.who.int/antimicrobial-resistance/interagency-coordination-group/IACG_Meeting_challenge_AMR_communication_to_collective_action_270718.pdf.

[5] Sriram A , Kalanxhi E , Kapoor G et al The State of the World’s Antibiotics 2021. Center for Disease Dynamics, Economics & Policy, 2021. https://cddep.org/publications/the-state-of-the-worlds-antibiotic-in-2021/.

[6] Dagnall N , Drinkwater KG , Denovan A et al Bridging the gap between UK Government strategic narratives and public opinion/behavior: lessons from COVID-19. Front Commun 2020; 5: 71.

[7] WHO. Global Action Plan on Antimicrobial Resistance. 2015. https://www.who.int/publications/i/item/9789241509763.

[8] Wellcome Trust. Reframing Resistance: How to Communicate About Antimicrobial Resistance Effectively. 2019. https://wellcome.org/reports/reframing-antimicrobial-resistance-antibiotic-resistance.

[9] OMS. Antibiotic Resistance: Multi-Country Public Awareness Survey. WHO, 2015. http://apps.who.int/iris/bitstream/10665/194460/1/9789241509817_eng.pdf?ua=1. 10.3390/antibiotics14060599 PMC12189553 40558189

[10] Collins LC , Jaspal R , Nerlich B. Who or what has agency in the discussion of antimicrobial resistance in UK news media (2010–2015)? A transitivity analysis. Health (London) 2018; 22: 521–40. 28637360 10.1177/1363459317715777

[11] Wernli D , Jørgensen PS , Morel CM et al Mapping global policy discourse on antimicrobial resistance. BMJ Glob Health 2017; 2: e000378. 10.1136/bmjgh-2017-000378 PMC5717922 29225939

[12] WHO. Monitoring Global Progress on Addressing Antimicrobial Resistance. 2018. https://apps.who.int/iris/handle/10665/273128.

[13] Iossa G , White PCL. The natural environment: a critical missing link in national action plans on antimicrobial resistance. Bull World Health Organ 2018; 96: 858–60. 30505034 10.2471/BLT.18.210898 PMC6249705

[14] Essack SY. Environment: the neglected component of the One Health triad. Lancet Planet Health 2018; 2: e238–9. 29880152 10.1016/S2542-5196(18)30124-4 PMC7129022

[15] Kamenshchikova A , Wolffs PFG , Hoebe CJPA et al Anthropocentric framings of One Health: an analysis of international antimicrobial resistance policy documents. Crit Public Health 2021; 31: 306–15.

[16] Huttner B , Saam M , Moja L et al How to improve antibiotic awareness campaigns: findings of a WHO global survey. BMJ Glob Health 2019; 4: e001239. 10.1136/bmjgh-2018-001239 PMC6528771 31179029

[17] Will CM. The problem and the productivity of ignorance: public health campaigns on antibiotic stewardship. Sociol Rev 2020; 68: 55–76.

[18] PHE. Keep Antibiotics Working. https://campaignresources.phe.gov.uk/resources/campaigns/58-keep-antibiotics-working.

[19] RUMA. Responsible Use of Antibiotics in Farm Animals – General Guidance, 2019. https://www.ruma.org.uk/ruma-information-note-on-antibiotics-and-the-responsible-use-of-antibiotics-in-farm-animals/.

[20] Broom A , Kenny K , Prainsack B et al Antimicrobial resistance as a problem of values? Views from three continents. Crit Public Health 2020; doi:10.1080/09581596.2020.1725444.

[21] Hinchliffe S , Butcher A , Rahman MM. The AMR problem: demanding economies, biological margins, and co-producing alternative strategies. Palgrave Commun 2018; 4: 142.

[22] Rochefort D , Cobb R. The Politics of Policy Definition: Shaping the Policy Agenda. University Press of Kansas, 1994.

[23] Walker S. Effective antimicrobial resistance communication: the role of information design. Palgrave Commun 2019; 5: 24.

[24] Matthews-King A. Drug Pollution in Rivers Reaching Damaging Levels for Animals and Ecosystems, Scientists Warn. The Independent, 2019. https://www.independent.co.uk/climate-change/news/drug-pollution-in-rivers-reaching-damaging-levels-for-animals-and-ecosystems-scientists-warn-a8792566.html.

[25] Harvey F. Farm Animals Antibiotics Data Raises Post-Brexit Trade Fears. The Guardian, 2020. https://www.theguardian.com/environment/2020/dec/01/farm-animals-antibiotics-data-raises-post-brexit-trade-fears.

[26] Brügger A , Dessai S , Devine-Wright P et al Psychological responses to the proximity of climate change. Nat Clim Chang 2015; 5: 1031–7.

[27] Collignon P , Beggs JJ , Walsh TR et al Anthropological and socioeconomic factors contributing to global antimicrobial resistance: a univariate and multivariable analysis. Lancet Planet Health 2018; 2: e398–405. 30177008 10.1016/S2542-5196(18)30186-4

[28] Collignon P , Beggs JJ. Socioeconomic enablers for contagion: factors impelling the antimicrobial resistance epidemic. Antibiotics (Basel) 2019; 8: 86. 10.3390/antibiotics8030086 PMC6784211 31261988

[29] Subbiah M , Caudell MA , Mair C et al Antimicrobial resistant enteric bacteria are widely distributed amongst people, animals and the environment in Tanzania. Nat Commun 2020; 11: 228. 31932601 10.1038/s41467-019-13995-5 PMC6957491

[30] Pearson M , Chandler C. Knowing antimicrobial resistance in practice: a multi-country qualitative study with human and animal healthcare professionals. Glob Health Action 2019; 12: 15–7. 10.1080/16549716.2019.1599560 PMC6703149 31294679

[31] United Nations Environment Programme. Frontiers 2017: Emerging Issues of Environmental Concern. https://www.unep.org/resources/frontiers-2017-emerging-issues-environmental-concern.

[32] Mendelson M , Balasegaram M , Jinks T et al Antibiotic resistance has a language problem. Nature 2017; 545: 23–5. 28470219 10.1038/545023a

[33] Krockow EM. *Nomen est omen*: why we need to rename ‘antimicrobial resistance’. JAC Antimicrob Resist 2020; 2: dlaa067. 34192252 10.1093/jacamr/dlaa067 PMC7454604

[34] Bushell S , Buisson GS , Workman M et al Strategic narratives in climate change: towards a unifying narrative to address the action gap on climate change. Energy Res Soc Sci 2017; 28: 39–49.

